# Successful treatment with bortezomib in combination with dexamethasone in a middle-aged male with idiopathic multicentric Castleman’s disease: A case report

**DOI:** 10.1515/med-2023-0763

**Published:** 2024-01-19

**Authors:** Hongling Li, Yang He, Yongying Wang, Mengwei Xu

**Affiliations:** Department of Oncology, Gansu Provincial Hospital, 204 West Donggang Road, 730000, Gansu, Lanzhou, China; College of Clinical Medicine, Ningxia Medical University, Yinchuan, China; First College of Clinical Medicine, Gansu University of Chinese Medicine, Lanzhou, China; Department of Pathology, Xijing Hospital, Fourth Military Medical University, Xi’an, China; Department of Oncology, Gansu Provincial Hospital, Gansu, Lanzhou, China

**Keywords:** idiopathic multicentric Castleman disease, bortezomib, salvage strategy, refractory, case report

## Abstract

Multicentric Castleman disease (MCD) is a heterogeneous, life-threatening disease. A subgroup of HIV-negative and HHV-8-negative MCD is defined as idiopathic MCD (iMCD) with a poor prognosis. Here we report an unusual case of a 47-year-old male patient with iMCD who experienced multiple treatment regimens such as chemotherapy, immunomodulatory therapy, and targeted therapy, all of which were considered ineffective. Subsequently, he was started on bortezomib in combination with dexamethasone for six cycles and he was in complete remission. The patient has survived nearly 13 years to date – the longest survival of any iMCD patient treated with bortezomib in combination with dexamethasone. Bortezomib combined with dexamethasone may be an effective salvage strategy for severe and refractory iMCD.

## 1 Introduction

Multicentric Castleman disease (MCD) is a heterogeneous group of chronic lymphoid tissue proliferative disorders characterized by enlarged lymph nodes. This group of diseases shares common histopathological features, including hyaline-vascular, plasma cell (PC), and hyaline-vascular plasma cell type [1,2]. MCD involves multiple lymph node sites, which is distinguished from unicentric Castleman (UCD), and often presents with systemic symptoms such as fever, night sweats, malaise, edema, anemia, and elevated C-reactive protein (CRP) and hypoproteinemia [3]. MCD can be further divided into idiopathic MCD (iMCD), human herpesvirus type 8 (HHV8)-associated MCD, and MCD with POEMS (polyneuropathy, organomegaly, endocrinopathy, M-protein, and skin changes) [1], where the etiology of HHV8-associated MCD is well-defined and effective treatments are available [4]. In contrast, the etiology and pathogenesis of HHV-8-negative iMCD have not been clearly defined to date. iMCD is widely believed to be driven by IL-6 signaling to develop the disease, but whether other signaling pathways and cytokines are involved remain unknown. iMCD is characterized by systemic symptoms, multiregional lymph node involvement, and the typical histopathological manifestations of Castleman’s disease. Autoimmune-related symptoms are more frequently observed in iMCD than in HHV8-related MCD and UCD, mainly including arthritis or renal insufficiency with proteinuria [5]. CD3+ lymphocytes are significantly higher in the lymph nodes and CD19+/CD5+ lymphocytes appear less frequently in patients with iMCD compared to patients with UCD [6]. In addition, unlike HHV8-associated MCD, iMCD is not correlated with plasmacytoid lymphoma and Kaposi’s sarcoma [7]. iMCD has a low incidence, with a prevalence of only 1,000–1,500 cases in the United States and a slightly higher incidence in Asian countries [8]. Consequently, clinical awareness and management of the disease are lacking, and prospective clinical trials are difficult to conduct, preventing the diagnosis and treatment of the disease from gaining momentum. The diversity of its clinical manifestations makes the diagnosis of iMCD more challenging, where clinical features such as PC variant subtype, age >40 years, TAFRO subtype, organ dysfunction, and inflammation levels are significantly associated with poor prognosis [9]. In addition, iMCD patients have a variable response to treatment, with only 10–20% of iMCD patients receiving glucocorticoids and chemotherapy achieving CR, and those who achieve CR often experience disease relapse within 1–2 years [9]. Furthermore, the overall prognosis of such patients is poor, with 5-year survival rates of only 51–77% [2]. In continuous exploration and conclusion, the first international consensus on iMCD treatment was published only in 2018, continuous clinical practice is still needed to evaluate the optimal treatment options applicable to each patient.

Currently, siltuximab and tocilizumab (an anti-IL-6 monoclonal antibody) are first-line agents for the treatment of iMCD and have been proven to be safe and effective in multiple practices. In a clinical trial, van Rhee et al. demonstrated that siltuximab significantly prolonged the life expectancy of patients with iMCD, with 34% achieving remission status, and that the incidence of adverse events associated with the drug was no higher than in the control group. It also reported no treatment-related deaths, so the drug can be considered as a safe agent [10,11]. Tocilizumab is a more personalized agent available to treat not only the disease itself but also a variety of complications in MCD patients such as thrombocytopenia, ascites, renal failure, and myelofibrosis as well as cardiomyopathy [11–13]. Rituximab, which targets B lymphocytes, is used in the first-line treatment of HHV8-MCD. It is also used in combination with conventional chemotherapeutic agents (such as the R-CHOP) in the treatment of iMCD patients when anti-IL-6 agents are ineffective. The selective proteasome inhibitor bortezomib is only available as a second-line treatment option and beyond, but also offers hope for a proportion of iMCD patients [1]. Bortezomib in combination with dexamethasone, characterized by ablation of a highly activated immune system and blocking cytokine storm, is a second-line and above treatment option in patients with the worsening disease or in those who do not respond to siltuximab [5]. Of these, bortezomib is a selective and reversible class of 26S proteasome inhibitors, a drug that is thought to act by blocking the production of nuclear factor-κB-dependent cytokines, such as IL-6 [14–16], which is essential in the pathogenesis of iMCD. In a prospective study [17], the bortezomib–cyclophosphamide–dexamethasone regimen has been well demonstrated for its efficacy and safety in patients with relapsed and refractory (R/R) iMCD, and such multidrug combination chemotherapy regimens may provide an additional treatment option for some patients with iMCD. Here we report an unusual case of a 47-year-old male with severe iMCD, characterized by a series of complications including anemia, pleural and peritoneal effusions, multi-organ failure, and pulmonary infections, which was successfully treated with bortezomib in combination with dexamethasone and achieved long-term survival.

## 2 Case presentation

**Figure 1:**
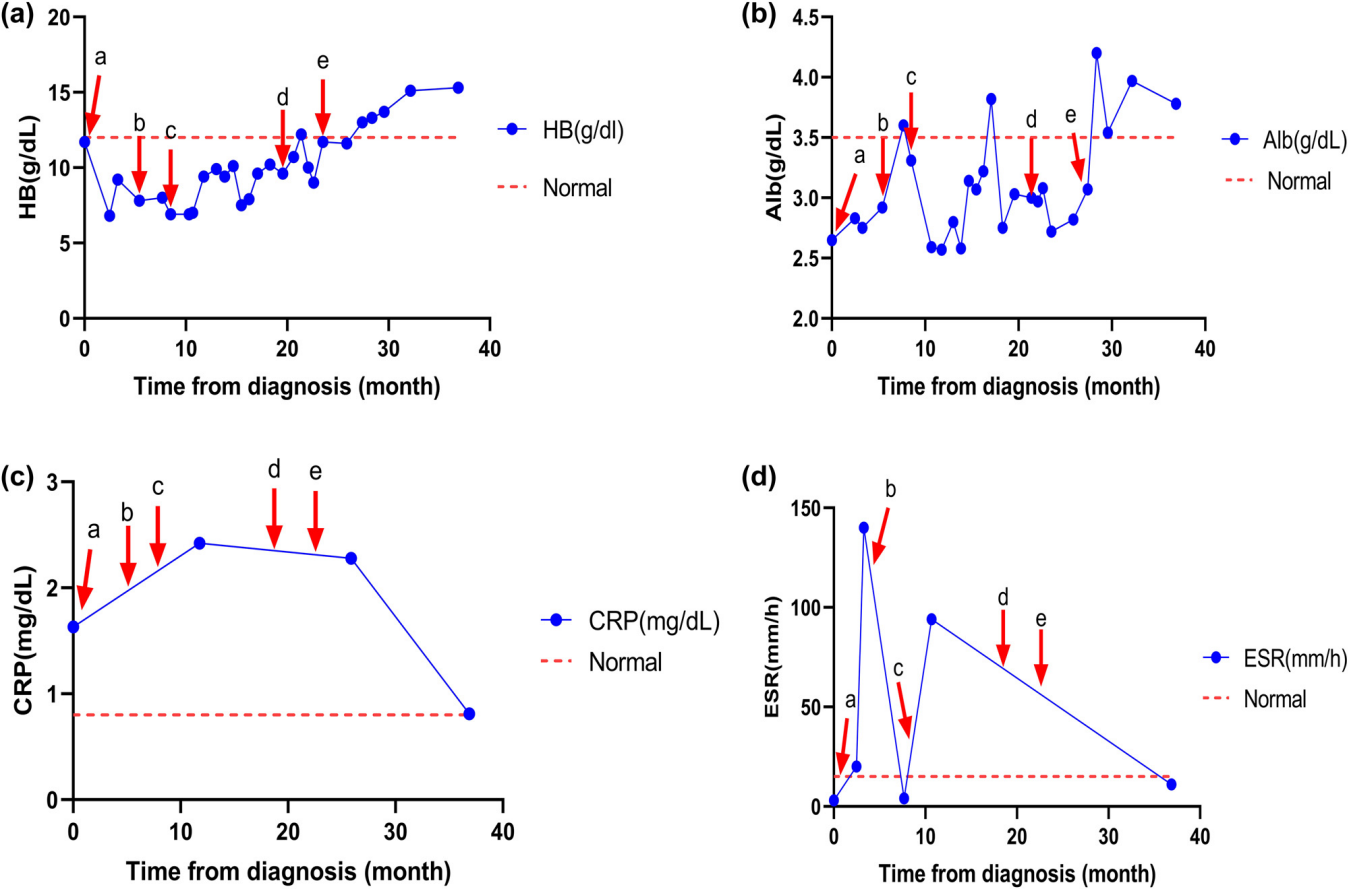
Representative laboratory data prior to and after application of different treatment regimens. Hemoglobin (a), albumin (b), CRP (c) levels, and ESR (d) improved with bortezomib combined with dexamethasone. (a: cyclophosphamide + vincristine + prednisone; b: cyclophosphamide + doxorubicin + vincristine + prednisone; c: interferon A + thalidomide; d: rituximab + interferon A + thalidomide; e: bortezomib + dexamethasone. Arrows represent the time of initiation of each treatment regimen.).

**Figure 2:**
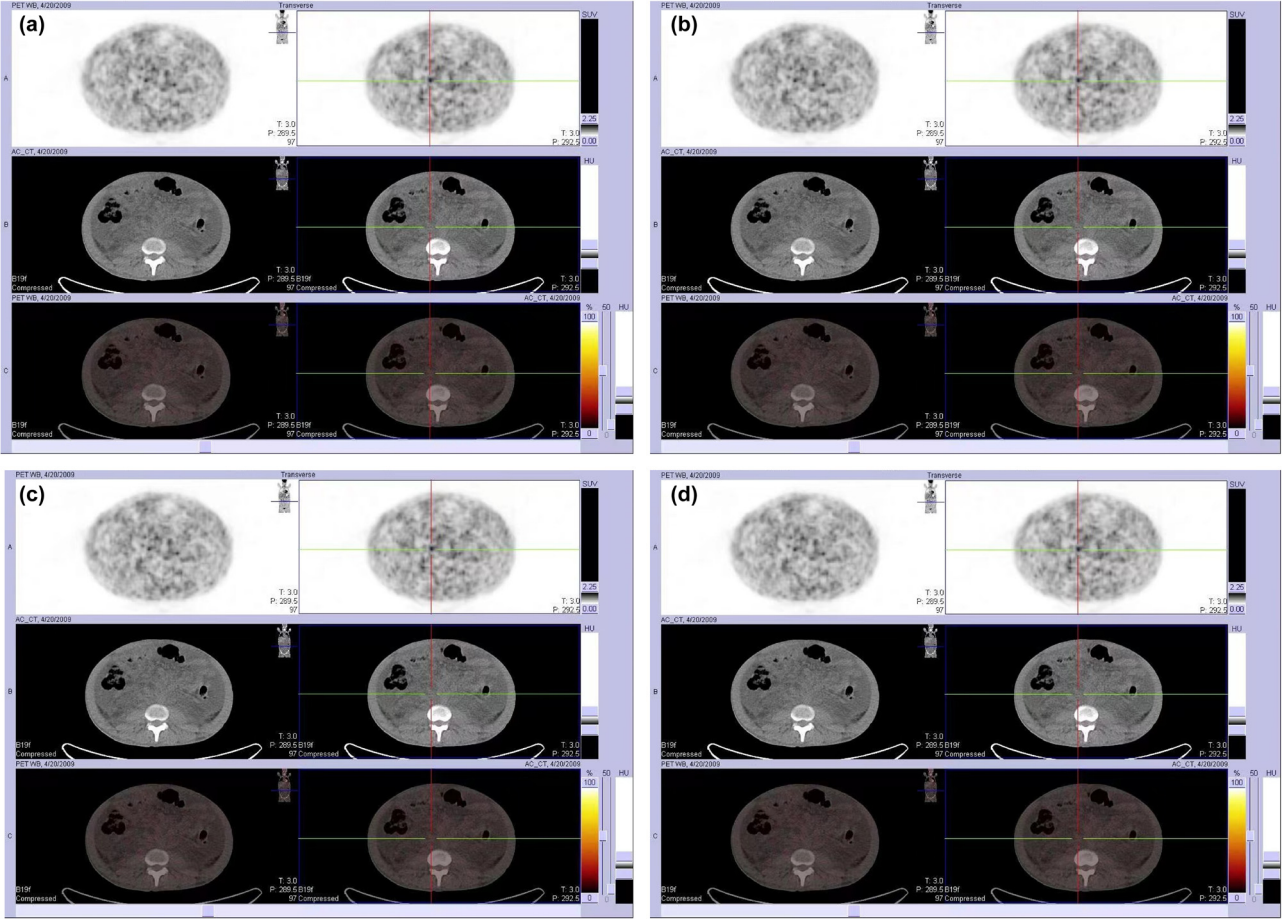
Fluorodeoxyglucose (FDG)-PET/CT scan at initial presentation revealed a mild abnormal accumulation of FDG in bilateral pleural effusion, as well as ascites, and multiple small lymph nodes (located behind the 12th thoracic and 4th lumbar vertebrae, with a diameter between 4 and 9 mm) (maximum standard uptake value: 1.8–2.2).

**Figure 3:**
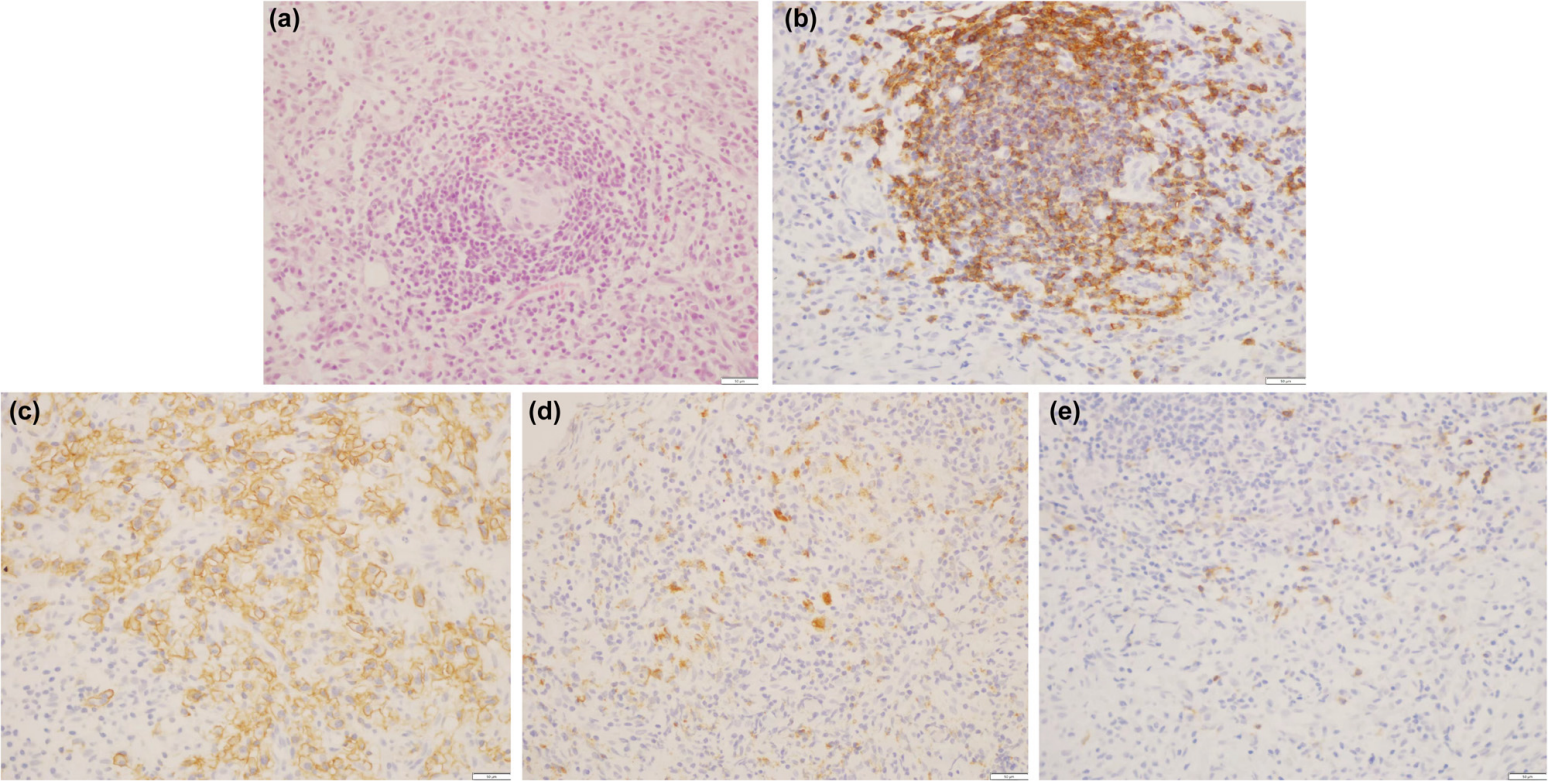
Lymph node biopsy showed multicentric Castleman’s disease of PC type. (a) Several lymphatic follicles were detected under light microscopy. The surrounding lymph nodes are arranged in concentric circles. The germinal center is atrophic, with a large number of PCs and a few lymphatic infiltrates in the interfollicles (hematoxylin and eosin, ×400). (b) Immunohistochemical (IHC) staining of cells by CD20 antibody, which shows positive follicular areas. (c) IHC staining CD138 PC expression is positive and shows cell membrane staining. (d) IHC staining CD3 and (e) CD68 interfollicular areas are scattered positive (IHC staining, ×20).

A 47-year-old Chinese male was admitted to our hospital in April 2009. He reported bilateral lower limb edema with malaise for over 1 year, followed by a gradual onset of abdominal distention that lasted for 2 months. Physical examination revealed red papules on his face and an enlarged spleen with mild induration. No enlarged superficial lymph nodes were palpated and the rest of the medical history was unremarkable. His laboratory data revealed reduced levels of hemoglobin (11.7 g/dL; normal range, 12.0–16.0 g/dL) and albumin (2.65 g/dL; normal range, 3.5–5.5 g/dL), and elevated serum CRP levels (1.63 mg/dL; normal range, 0.0–0.8 mg/dL) (Figure 1). An abdominal computed tomography (CT) scan revealed hepatomegaly, splenomegaly, and effusion in the chest and abdominal cavity. Positron emission tomography-computed tomography (PET-CT) scan showed multiple enlarged retroperitoneal lymph nodes (maximum diameter 4 × 9 mm) (Figure 2). Subsequently, a bone marrow aspiration was performed and there were no significant abnormalities. As malignancy could not be completely excluded, a puncture biopsy of the enlarged abdominal lymph nodes was performed and the pathology was consistent with multicentric Castleman’s disease, PC type (Figure 3). Further examination of the patient’s peripheral blood was negative for HHV8 and HIV.

After diagnosis in April 2009, the patient was initially treated with the COP (cyclophosphamide, vincristine, and prednisone) chemotherapy regimen, which was replaced by the CHOP (cyclophosphamide, doxorubicin, vincristine, and prednisone) regimen in September 2009 due to lack of improvement in his symptoms. During the treatment period, he developed fever, cough, and worsening edema in his lower limbs, his hemoglobin dropped to 7.8 g/dL and white blood cells dropped to 2.5 × 109/L, which precluded him receiving further chemotherapy. Subsequently, the patient’s condition deteriorated dramatically, with a weight loss from 65 to 35 kg, a rapid increase in ascites and pleural effusion, and numerous episodes of anemia requiring blood transfusions with an Eastern Cooperative Oncology Group (ECOG) score of 4. In December 2009, the patient received 11 months of immunomodulatory therapy, mainly interferon A and thalidomide (100 mg/day), after which his general condition improved. Regrettably, the patient still had symptoms of anemia, no reduction in the enlarged retroperitoneal lymph nodes on CT scan, and the disease was still slowly progressing.

Hence, after discussing the treatment plan with the patient in March 2011, he received a regimen of bortezomib (1.3 mg/m^2^) combined with dexamethasone on Days 1, 4, 8, and 11, and repeated on Day 22. However, the patient had to interrupt his treatment after three cycles owing to the need for endoscopic retrograde cholangiopancreatography for his gallbladder stones. After surgery, the patient continued to receive three cycles of chemotherapy. At the end of treatment, a health assessment in April 2012 showed that the patient’s HB level and albumin level increased to 15.3 and 3.97 g/dL, respectively, and CRP and erythrocyte sedimentation rate (ESR) decreased to 0.809 mg/dL and 11 mm/h, respectively. His enlarged liver and spleen returned to normal size and the retroperitoneal lymph nodes were further reduced on CT images, with an ECOG score of 1. In general, the patient has well tolerated the medication with no adverse events during its administration. At the latest follow-up visit in February 2022, he had no recurrence of his disease and has returned to normal life and work.


**Informed consent:** Informed consent has been obtained from the patient.

## 3 Discussion

Until now, iMCD has been known for over 10 years, but its etiology and pathogenesis remain elucidated [1]. Various studies have shown that overproduction of cytokines (IL-6 and vascular endothelial growth factor [VEGF]), viral infections other than HHV-8, and immune diseases play a critical role in the pathogenesis of some cases [3,18]. Targeting different treatment regimens for the cause of the disease has also been refined, including combination chemotherapy, B-cell depleting agents, immunomodulators, and anti-IL-6 targeting monoclonal antibodies. Owing to the heterogeneous and rare nature of iMCD, however, there is variability in the response to treatment in different individuals [19].

According to the consensus iMCD treatment guidelines [5] published in 2018, our case was identified as severe iMCD with a PC subtype of pathology and an unfavorable prognosis. With respect to the choice of the best treatment for this patient was a clinical challenge we faced. The results of a study [20] showed that CHOP regimen had an overall response rate of approximately 90% in iMCD, with 50% showing complete remission. In another prospective study [21], of the ten patients with iMCD treated with COP or CHOP, one had a complete remission and six had a partial remission, which was a more significant treatment effect. In our case, based on our experience with non-Hodgkin’s lymphoma, the patient was treated sequentially with a chemotherapy regimen of COP and CHOP. However, he only obtained a treatment response of disease stabilization and we abandoned this regimen. Subsequently, considering that iMCD may be due to an unexplained viral infection, the patient was treated with antiviral and immunomodulatory therapy. Interferon A and thalidomide, not only have immunomodulatory, antiviral, and anti-angiogenic effects but also have the property of inhibiting the production of the pro-inflammatory cytokines IL-1, IL-6, and IL-12 [19,22]. Unfortunately, only a partial response was achieved in our patients. The anti-CD20 drug rituximab, which is considered to be therapeutic that can cause lasting remission [23], did not yield any positive response in our patients. Siltuximab, an anti-IL-6 targeting monoclonal antibody, is currently the first-line therapy of choice for iMCD, but we did not have access to this drug.

Ultimately, the patient was started on bortezomib in combination with dexamethasone for a total of six cycles. Bortezomib is the first selective proteasome inhibitor to enter clinical practice and is currently used in the treatment of a variety of hematological disorders. One of the most successful applications is the treatment of adult multiple myeloma, with significant anti-tumor activity in both untreated and refractory/recurrent multiple myeloma (MM) patients [24–26]. The drug inhibits the growth of MM tumor cells by inhibiting the activity of NF-κB and blocking the production of cytokines such as IL-6 and VEGF. In combination with dexamethasone, this inhibitory effect with synergy will play an even greater role [17,27]. IL-6 is also considered to be the main pathogenic mechanism of iMCD, causing B symptoms in the organism and also inhibiting albumin synthesis, which leads to pleural and abdominal effusions in patients [23]. To this extent, this may explain the initial clinical manifestations of our patient. IL-6 is thought to be intensively associated with the pathogenesis of PC tumors. Overexpression of IL-6 in IL-6 transgenic mice or introduction of IL-6-containing retroviruses into stem cells will induce iMCD [28], and monoclonal antibodies against IL-6 or IL-6 receptors developed at this stage against this target may attenuate the clinical manifestations associated with iMCD patients. The blockade of IL-6 by bortezomib may be part of what allowed our patients to achieve a good outcome.

In the iMCD PC subtype, IL-6 produced by lymph nodes may induce paracrine secretion of VEGF from PCs and vascular proliferation in the intercapsular region of the affected lymph nodes [27]. We speculate that bortezomib may exert a therapeutic effect in iMCD by inhibiting the above processes. We speculate that inhibition of the above processes by bortezomib is another reason for the therapeutic effect.

Nevertheless, about half of the iMCD patients failed to respond to IL-6 inhibition, suggesting that there may be other unknown cytokines and signaling pathways involved in the development of the disease [1]. There are other hypotheses proposed for the disease, partly iMCD patients overlap with autoimmune diseases [29], yet the patients we reported had no significant abnormalities in autoantibodies and therefore could not be tested from this aspect. The hypothesis that tumor cells drive iMCD is also interesting [29], but additional data are required to support this opinion.

Previously, cases of bortezomib combined with dexamethasone successfully treating MCD combined with myeloma have been reported, achieving excellent partial remission [15,30]. Sobas et al. [19] reported that bortezomib was effective in refractory MCD associated with POEMS. This suggests that this combination therapy may be a boon for patients with severe, refractory iMCD and that further clinical studies could be conducted to assess its potential therapeutic value.

## References

[1] Dispenzieri A, Fajgenbaum DC. Overview of Castleman disease. Blood. 2020 Apr;135(16):1353–64. 10.1182/blood.2019000931.32106302

[2] Zhang L, Zhao AL, Duan MH, Li ZY, Cao XX, Feng J, et al. Phase 2 study using oral thalidomide–cyclophosphamide–prednisone for idiopathic multicentric Castleman disease. Blood. 2019 Apr;133(16):1720–8. Epub 2019 Feb 13 10.1182/blood-2018-11-884577. 30760451

[3] Fajgenbaum DC, Uldrick TS, Bagg A, Frank D, Wu D, Srkalovic G, et al. International, evidence-based consensus diagnostic criteria for HHV-8-negative/idiopathic multicentric Castleman disease. Blood. 2017 Mar;129(12):1646–57. Epub 2017 Jan 13. 10.1182/blood-2016-10-746933. PMC5364342 28087540

[4] Uldrick TS, Polizzotto MN, Aleman K, Wyvill KM, Marshall V, Whitby D, et al. Rituximab plus liposomal doxorubicin in HIV-infected patients with KSHV-associated multicentric Castleman disease. Blood. 2014 Dec;124(24):3544–52. Epub 2014 Oct 20. 10.1182/blood-2014-07-586800. PMC4256906 25331113

[5] van Rhee F, Voorhees P, Dispenzieri A, Fosså A, Srkalovic G, Ide M, et al. International, evidence-based consensus treatment guidelines for idiopathic multicentric Castleman disease. Blood. 2018 Nov;132(20):2115–24. Epub 2018 Sep 4. 10.1182/blood-2018-07-862334. PMC6238190 30181172

[6] Yu L, Tu M, Cortes J, Xu-Monette ZY, Miranda RN, Zhang J, et al. Clinical and pathological characteristics of HIV- and HHV-8-negative Castleman disease. Blood . 2017 Mar;129(12):1658–68. Epub 2017 Jan 18. 10.1182/blood-2016-11-748855. PMC5364343 28100459

[7] Arenas DJ, Floess K, Kobrin D, Pai RL, Srkalovic MB, Tamakloe MA, et al. Increased mTOR activation in idiopathic multicentric Castleman disease. Blood. 2020 May;135(19):1673–84. 10.1182/blood.2019002792. PMC7205815 32206779

[8] Simpson D. Epidemiology of castleman disease. Hematol Oncol Clin North Am. 2018 Feb;32(1):1–10. 10.1016/j.hoc.2017.09.001. 29157611

[9] Yu L, Shi M, Cai Q, Strati P, Hagemeister F, Zhai Q, et al. A novel predictive model for idiopathic multicentric castleman disease: the International Castleman Disease Consortium Study. Oncologist. 2020 Nov;25(11):963–73. Epub 2020 Sep 18. 10.1634/theoncologist.2019-0986. PMC7648372 32852137

[10] van Rhee F, Wong RS, Munshi N, Rossi JF, Ke XY, Fosså A, et al. Siltuximab for multicentric Castleman’s disease: a randomised, double-blind, placebo-controlled trial. Lancet Oncol. 2014 Aug;15(9):966–74. Epub 2014 Jul 17. Erratum in: Lancet Oncol. 2014 Sep;15(10):417. 10.1016/S1470-2045(14)70319-5. 25042199

[11] Kapriniotis K, Lampridis S, Mitsos S, Patrini D, Lawrence DR, Panagiotopoulos N. Biologic agents in the treatment of multicentric castleman disease. Turk Thorac J. 2018 Oct;19(4):220–5. Epub 2018 Oct 1. 10.5152/TurkThoracJ.2018.18066. PMC6196909 30455994

[12] Kawabata H, Kotani S, Matsumura Y, Kondo T, Katsurada T, Haga H, et al. Successful treatment of a patient with multicentric Castleman’s disease who presented with thrombocytopenia, ascites, renal failure and myelofibrosis using tocilizumab, an anti-interleukin-6 receptor antibody. Intern Med. 2013;52(13):1503–7. Epub 2013 Jul 1. 10.2169/internalmedicine.52.9482. 23812199

[13] Man L, Goudar RK. Reversal of cardiomyopathy with tocilizumab in a case of HIV-negative Castleman’s disease. Eur J Haematol. 2013 Sep;91(3):273–6. Epub 2013 Jul 22. 10.1111/ejh.12161. 23786462

[14] Robak P, Robak T. Bortezomib for the treatment of hematologic malignancies: 15 years later. Drugs R D. 2019 Jun;19(2):73–92. 10.1007/s40268-019-0269-9. PMC6544598 30993606

[15] Yuan ZG, Dun XY, Li YH, Hou J. Treatment of multicentric Castleman’s disease accompanying multiple myeloma with bortezomib: a case report. J Hematol Oncol. 2009 Apr;2:19. 10.1186/1756-8722-2-19. PMC2686727 19400935

[16] Rajkumar SV, Richardson PG, Hideshima T, Anderson KC. Proteasome inhibition as a novel therapeutic target in human cancer. J Clin Oncol. 2005 Jan;23(3):630–9. 10.1200/JCO.2005.11.030. 15659509

[17] Zhang L, Zhang MY, Cao XX, Zhou DB, Fajgenbaum DC, Dong YJ, et al. A prospective, multicenter study of bortezomib, cyclophosphamide, and dexamethasone in relapsed/refractory iMCD. Leuk Lymphoma. 2022 Mar;63(3):618–26. Epub 2022 Jan 31. 10.1080/10428194.2021.1999437. 35100929

[18] Casper C. The aetiology and management of Castleman disease at 50 years: translating pathophysiology to patient care. Br J Haematol. 2005 Apr;129(1):3–17. 10.1111/j.1365-2141.2004.05311.x. 15801951

[19] Sobas MA, Alonso Vence N, Diaz Arias J, Bendaña Lopez A, Fraga Rodriguez M, Bello Lopez JL. Efficacy of bortezomib in refractory form of multicentric Castleman disease associated to poems syndrome (MCD-POEMS variant). Ann Hematol. 2010 Feb;89(2):217–9. Epub 2009 Jul 28. 10.1007/s00277-009-0795-6. 19636554

[20] Ocio EM, Sanchez-Guijo FM, Diez-Campelo M, Castilla C, Blanco OJ, Caballero D, et al. Efficacy of rituximab in an aggressive form of multicentric Castleman disease associated with immune phenomena. Am J Hematol. 2005 Apr;78(4):302–5. 10.1002/ajh.20283. 15795923

[21] Zhu SH, Yu YH, Zhang Y, Sun JJ, Han DL, Li J. Clinical features and outcome of patients with HIV-negative multicentric Castleman’s disease treated with combination chemotherapy: a report on 10 patients. Med Oncol. 2013 Mar;30(1):492. 10.1007/s12032-013-0492-0. Epub 2013 Feb 12. 23400962

[22] Muller GW, Corral LG, Shire MG, Wang H, Moreira A, Kaplan G, et al. Structural modifications of thalidomide produce analogs with enhanced tumor necrosis factor inhibitory activity. J Med Chem. 1996 Aug;39(17):3238–40. 10.1021/jm9603328. 8765505

[23] van Rhee F, Greenway A, Stone K. Treatment of idiopathic Castleman disease. Hematol Oncol Clin North Am. 2018 Feb;32(1):89–106. 10.1016/j.hoc.2017.09.008. 29157622

[24] Citrin R, Foster JB, Teachey DT. The role of proteasome inhibition in the treatment of malignant and non-malignant hematologic disorders. Expert Rev Hematol. 2016 Sep;9(9):873–89. Epub 2016 Aug 5. 10.1080/17474086.2016.1216311. 27447436

[25] Kane RC, Farrell AT, Sridhara R, Pazdur R. United States Food and Drug Administration approval summary: bortezomib for the treatment of progressive multiple myeloma after one prior therapy. Clin Cancer Res. 2006 May;12(10):2955–60. 10.1158/1078-0432.CCR-06-0170. 16707588

[26] Palumbo A, Chanan-Khan A, Weisel K, Nooka AK, Masszi T, Beksac M, et al. Daratumumab, bortezomib, and dexamethasone for multiple myeloma. N Engl J Med. 2016 Aug;375(8):754–66. 10.1056/NEJMoa1606038. 27557302

[27] Nishi J, Maruyama I. Increased expression of vascular endothelial growth factor (VEGF) in Castleman’s disease: proposed pathomechanism of vascular proliferation in the affected lymph node. Leuk Lymphoma. 2000 Jul;38(3–4):387–94. 10.3109/10428190009087030. 10830746

[28] Brandt SJ, Bodine DM, Dunbar CE, Nienhuis AW. Retroviral-mediated transfer of interleukin-6 into hematopoietic cells of mice results in a syndrome resembling Castleman’s disease. Curr Top Microbiol Immunol. 1990;166:37–41. 10.1007/978-3-642-75889-8_5. 2073814

[29] Liu AY, Nabel CS, Finkelman BS, Ruth JR, Kurzrock R, van Rhee F, et al. Idiopathic multicentric Castleman’s disease: a systematic literature review. Lancet Haematol. 2016 Apr;3(4):e163–75. Epub 2016 Mar 17. 10.1016/S2352-3026(16)00006-5. 27063975

[30] Khan AA, Siraj F, Bhargava M, Aggarwal S. Successful treatment of multicentric Castleman’s disease accompanying myeloma with bortezomib. BMJ Case Rep. 2012 Dec;2012:bcr2012007646. 10.1136/bcr-2012-007646. PMC4543972 23264156

